# Functionalization of 7-Hydroxy-pyranoflavylium: Synthesis of New Dyes with Extended Chromatic Stability

**DOI:** 10.3390/molecules27217351

**Published:** 2022-10-29

**Authors:** Ana Rita Pereira, Victor de Freitas, Nuno Mateus, Joana Oliveira

**Affiliations:** Laboratório Associado para a Química Verde—REQUIMTE, Departamento de Química e Bioquímica, Faculdade de Ciências, Universidade do Porto, Rua do Campo Alegre, 687, 4169-007 Porto, Portugal

**Keywords:** 7-hydroxy-pyranoflavylium, 1-(3-dimethylaminopropyl)-3-ethylcarbodiimide hydrochloride, ester bond, coupling chemistry, cinnamic acids

## Abstract

This work reports the functionalization of pyranoflavyliums pigment using 1-(3-dimethylaminopropyl)-3-ethylcarbodiimide hydrochloride coupling chemistry. Four cinnamic acids were used to establish an ester bond with the hydroxyl group of the pyranoflavylium, namely 4-dimethylamino-, 4-amino-, 4-bromo-, and trans-cinnamic acids. The experimental condition, namely the molar ratios, solvent, and reaction time, were adjusted to obtain higher reaction yields in a reduced period. Excellent reaction yields of 68%, 85%, 94%, and 99% were achieved for 4-amino, trans-, 4-bromo, and 4-dimethylamino pyranoflavylium cinnamates, respectively. The structure of the functionalized pigments was fully clarified using one-dimensional (1H) and two-dimensional (COSY, HSQC, and HMBC) NMR experiments and HRSM analysis. Regardless of the type of functionalization, the UV-Visible spectrum showed a bathochromic shift (red region) on the maximum absorption wavelength and the absence of acid-base reactions throughout a broad pH range in comparison to the pyranoflavylium precursor. This work offers a valuable environmentally friendly, quick, and straightforward alternative to flavylium compounds’ challenging and labor-intensive functionalization, resulting in novel dyes with higher stability and dissimilar chromatic features.

## 1. Introduction

Over the last years, several classes of natural and synthetic flavylium-based dyes have been described in the literature, including anthocyanins [1,2], 3-deoxyanthocyanins [3,4], A- and B-type vitisins [5,6,7], methylpyranoanthocyanins [8], oxovitisins [9], and other pyranoanthocyanins [10,11,12]. Some of them are present in large amounts in the plant kingdom, namely anthocyanins, while others result from the reaction of these natural pigments with other organic molecules in processed matrices such as fruit juices and red wines [5,6,7]. Considering the singular properties and the wide range of applications of these molecules, extensive works reporting all the synthetic and enzymatic strategies and mechanisms have been performed over the years to obtain different classes of flavylium-based compounds [13,14]. On the other hand, other authors are focused on subjects such as the molecular and supramolecular structures of natural anthocyanin-derived pigments and the copigmentation effect of these compounds [15,16]. Bioinspired synthetic flavylium compounds have been a major focus of some research groups, resulting from acidic aldolic condensation reactions between a salicylaldehyde-derivative and an acetophenone [14,17,18,19,20]. Amino-based flavylium dyes contribute to a palette of different shades of blue derived from the flavylium core due to a bathochromic shift associated with a higher absorption in the red region by the inclusion of N-atoms with electron-donating capacity improving the π-conjugation [17]. However, some of them have significant solubility and stability issues that restrict their use in certain applications. The existence of hydroxyl groups is another crucial factor that influences the occurrence of various species as a result of the pH variation, which promotes proton transfer reactions. To overcome this, the present work proposes the functionalization of a *p*-dimethylamino-phenyl-7-hydroxyl-pyranoflavylium dye with different cinnamic acid derivatives via an ester bond, giving new functionality to the hydroxyl group and reducing acid-base reactions at a wide pH range. When compared to other molecules, the chemical modification of flavylium and/or derivatives remains a constant challenge. Electronic conjugation and delocalization properties around the oxonium moiety, and the occurrence of various dynamic equilibrium forms in solution, affect the reactivity and the availability of these compounds. Alkylation and acylation are the main functionalizations of anthocyanins reported in the literature, although the acylation with different fatty acids has been carried out using chemical [21] and enzymatic methodologies [22,23,24,25]. More recently, a green and sustainable alternative combining microwave-assisted synthesis and ionic liquids was proposed in comparison to the conventional heating and organic solvents [26]. Nevertheless, all these functionalizations were selective for the glucose primary hydroxyl group present in anthocyanins. The synthesis of bis-flavylium compounds is another strategy adopted to obtain functionalized compounds that involve a variety of reactional stages, reagents, solvents, and temperatures [27,28]. For this, acetophenone or aldehyde, one of the initiating reagents used in the synthesis of a flavylium, undergoes one or more reactions with nucleophilic and oxidizing agents, such as magnesium bromide and 2-iodoxybenzoic acid (IBX), to produce an intermediate product. The appropriate bis-flavylium compounds are then produced by the second reagent’s highly effective acid-catalyzed aldolic condensation [28,29]. The addition of halogens to the flavylium core’s ring A is another form of flavylium functionalization. As an example, the bromination of the aldehyde used in aldolic condensation to produce the flavylium compound is functionalized with a bromine atom. This intermediate product undergoes an acid-catalyzed aldolic condensation with acetophenone, after purification and characterization, to produce a variety of isomers that are then separated and further purified [29]. Due to the complexity of these chemicals, every method used is time-consuming and tedious.

This work reports a coupling reaction to functionalize a 7-hydroxyl pyranoflavylium dye in order to develop a cleaner, but equally effective, method for the functionalization of flavylium compounds. The process involves a single reaction step using EDC as a coupling agent between the various cinnamic acids and the previously synthesized pyranoflavylium pigment.

## 2. Results

### 2.1. Esterification of Amino-Based 7-Hydroxyl Pyranoflavylium

Amino-based flavylium dyes are bluish molecules with a maximum wavelength of around 550 nm. The precursor of *p*-dimethylamino-phenyl-7-hydroxyl-pyranoflavylium functionalized in this study has a single hydroxyl group on carbon C-7 of ring A, making it a good candidate for selective esterification with various compounds that results in a single reaction product. Because of their antioxidant, antibacterial, anticancer, neuroprotective, anti-inflammatory, and anti-diabetic characteristics, cinnamic acids derivatives are useful in chemical synthesis [30]. The direct esterification reaction between 7-hydroxy-pyranoflavylium and each cinnamic acid was studied and it was observed that, under the experimental conditions used, no ester product was formed (Table 1—entry 1). The involvement of the hydroxyl group in the electronic delocalization in 7-hydroxy-pyranoflavylium derivatives may be responsible for the lower reactivity of this group.

**Scheme 1:**
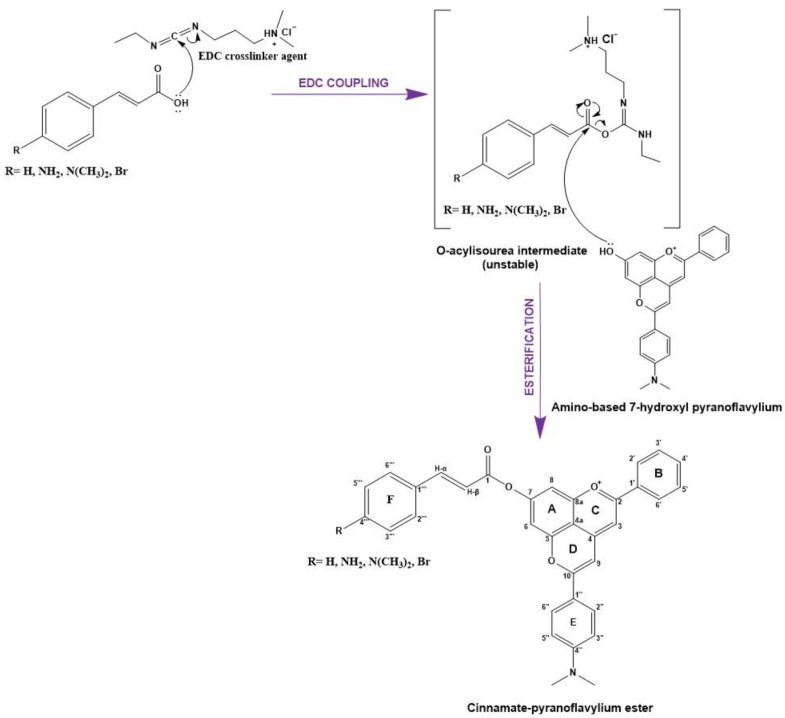
Mechanism of formation of the ester compounds from amino-based 7-hydroxyl pyranoflavylium and cinnamic acids derivatives using EDC coupling agent.

Consequently, the esterification of 7-hydroxyl-4″-(dimethylamino)-cinnamyl-10-pyranoflavylium with different cinnamic acids, using EDC as a coupling agent, is proposed. Carbodiimide compounds provide the most popular and versatile method for labeling or crosslinking to carboxylic acids. The most readily available and commonly used carbodiimides are the water-soluble EDC for aqueous crosslinking. Therefore, EDC and its by-product, isourea, are dissolved into the reaction medium, allowing easy purification of the crosslinked product by dialysis or ultrafiltration. In the simple mechanism of this coupling method, EDC first activates carboxyl groups of the cinnamic acid and forms an activated ester intermediate (*O*-acylisourea), which undergoes nucleophilic substitution in the presence of a strong nucleophile such as a hydroxyl group (-OH), yielding the respective ester product (Scheme 1).

**Figure 1:**
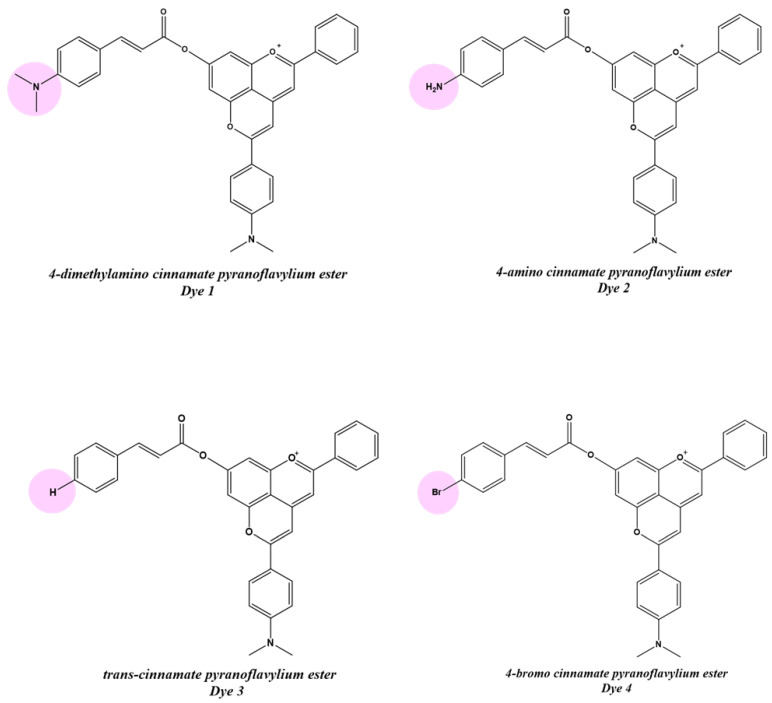
New dyes obtained by the esterification between cinnamic acids and a 7-hydroxyl pyranoflavylium derivative using the EDC coupling agent.

The optimization process was performed for all cinnamic derivatives. In the functionalization with 4-dimethylamino cinnamic acid, a reaction yield of 99% was achieved, and two synthetic routes can be followed: (i) the molar ratio of cinnamic acid derivative: EDC (1:5) and the molar ratio cinnamic acid derivative:pigment (10:1) in DMF at room temperature during 1 h under stirring or (ii) the molar ratio of cinnamic acid derivative:EDC (1:5) and the molar ratio cinnamic acid derivative: pigment (5:1) in DMF at room temperature during 20 h under stirring (Table 1, entry 6, and entry 11 (c), respectively). For 4-amino cinnamic acid, the maximum reaction yield obtained was around 69% when considering a molar ratio (1:1) between cinnamic acid derivative and EDC and a (10:1) molar ratio cinnamic acid derivative:pigment in constant stirring during 6 h (Table S1, entry 8 (b)). The lower yield obtained for the esterification with amino-cinnamic acid is due to the formation of a side-product in the reaction with the molecular ion at [M^+^] *m/z* 582, suggesting the nucleophilic addition of the secondary amine present in 4-amino pyranoflavylium cinnamate to the carbonyl group of dimethylformamide (Scheme S1). The esterification with 4-bromo cinnamic acid proved to be very promising, with a reaction yield of around 95% after 17 h of reaction (Table S1, entry 4 (b)), for a molar ratio of 1:2 and 10:1 between 4-bromo cinnamic acid:EDC and 4-bromo cinnamic acid:pyranoflavylium precursor, respectively. For the functionalization with *trans*-cinnamic acid, the reaction yield was around 85% for a molar ratio of 1:2 or 1:3 cinnamic acid derivative:EDC and 10:1 cinnamic acid derivative: 7-hydroxy-pyranoflavylium, to 17 h or 4 h of reaction, respectively (Table S1 entry 1 (b) and entry 2 (b), respectively). The residual *p*-dimethylamino-phenyl-7-hydroxyl-pyranoflavylium and other impurities, including EDC and urea byproducts, were eluted with methanol aqueous solution (up to 60%), while the ester products were eluted with 80–100% (*v*/*v*) methanol aqueous solution. The final fraction with the desired pigment was evaporated, frozen at −20 °C, and lyophilized. All pure compounds were stored at −20 °C for further analysis. In Figure 1, the structures of the new dyes obtained by coupling chemistry are presented.

### 2.2. Structural Characterization—LC-MS and NMR

Fractions containing the pure ester compound, and the respective precursor pigment, were analyzed by HRMS in the positive ion mode. For dyes 1, 2, 3, and 4, the full MS spectrum showed a molecular ion at [M^+^] *m/z* 555 (Figure S2), *m/z* 527 (Figure S3), *m/z* 512 (Figure S4), and *m/z* 591 (Figure S5), respectively, matching the structure of the new ester and suggesting the esterification at the hydroxyl group of the precursor *p*-dimethylamino-phenyl-7-hydroxyl-pyranoflavylium. In all ester products, the MS^2^ fragment at *m/z* 382 corresponds to the respective *p*-dimethylamino-phenyl-7-hydroxyl-pyranoflavylium.

To fully clarify the structure of the new esters, all of them were characterized by NMR spectroscopy using one-dimensional (^1^H NMR) and two-dimensional techniques (COSY, HSQC, HMBC) in the respective solvent. Using two-dimensional techniques (HSQC and HMBC), it was possible to provide the assignment of carbon resonances. Dye 1 (Figure S6): ^1^H NMR (600 MHz, Methanol-d4) δ 7.70 ppm (d, J = 6.5 Hz, H-2′, H-6′), 7.66 ppm (d, J = 16.0 Hz, H-α), 7.44 ppm (m, H-4′), 7.37 ppm (*, H-3′, H-5′), 7.37 ppm (*, H-2″, H-6″), 7.31 ppm (s, H-6), 7.17 ppm (s, H-8), 7.14 ppm (d, J = 7.4 Hz, H-2‴, H-6‴), 7.07 ppm (s, H-9), 6.97 ppm (s, H-3), 6.45 ppm (d, J = 7.4 Hz, H-3‴, H-5‴), 6.29 ppm (d, J = 7.8 Hz, H-3″, H-5″), 6.22 ppm (d, J = 16.0 Hz, H-β), 2.93 ppm (s, 4‴-H-N-CH3), 2.58 ppm (s, 4″-H-N-CH3). ^13^C NMR: δ 167.6 ppm (10D), 164.9 ppm (1-COO), 163.7 ppm (2C), 157.4 ppm (8aA), 155.5 ppm (4″E), 151.6 ppm (7A), 149.3 ppm (C-H-α), 133.7 ppm (4′B), 131.0 ppm (1″E), 130.6 ppm (2‴, 6‴F), 130.5 ppm (2″, 6″E), 130.4 ppm (1‴F), 129.1 ppm (3′, 5′B), 126.7 ppm (2′, 6′B), 126.4 ppm (1′B), 121.8 ppm (4‴F), 112.2 ppm (3″, 5″E), 111.8 ppm (3‴, 5‴F), 108.7 ppm (C-H-β), 108.7 ppm (4C, 4aA), 106.9 ppm (6A, 8A), 101.7 ppm (3C), 100.7 ppm (9D), 39.2 ppm (4‴-F-N-CH3), 38.6 ppm (4″-E-N-CH_3_). 

Dye 2 (Figure S7): ^1^H NMR (600 MHz, Methanol-d4) δ 7.87 ppm (d, J = 7.3 Hz, H-2′, H-6′), 7.74 ppm (d, J = 15.9 Hz, H-α), 7.67 ppm (d, J = 8.6 Hz, H-2″, H-6″), 7.52 ppm (m, H-4′), 7.46 ppm (d, J = 7.3 Hz, H-3′, H-5′), 7.42 ppm (s, H-6), 7.35 ppm (s, H-8), 7.31 ppm (d, J = 8.5 Hz, H-2‴, H-6‴), 7.28 ppm (s, H-9), 7.21 ppm (s, H-3), 6.75 ppm (d, J = 8.5 Hz, H-3‴, H-5‴), 6.53 ppm (d, J = 8.5 Hz, H-3″, H-5″), 6.37 ppm (d, J = 15.9 Hz, H-β), 2.82 ppm (s, H-N-CH3). ^13^C NMR: δ 168.1 ppm (10D), 164.6 ppm (1-COO), 164.0 ppm (2C), 156.9 ppm (8aA), 150.9 ppm (7A), 148.1 ppm (C-H-α), 147.7 ppm (4″E), 133.0 ppm (4′B), 130.4 ppm (1″E, 1‴E), 130.3 ppm (2″, 6″E and 2‴, 6‴F), 128.8 ppm (3′, 5′B), 126.7 ppm (2′, 6′B), 126.4 ppm (1′B), 121.8 ppm (4‴F), 115.9 ppm (3‴, 5‴F), 111.8 ppm (3″, 5″E), 109 ppm (C-H-β), 108.7 ppm (4C, 4aA), 106.2 ppm (6A), 106.2 ppm (8A), 101.4 ppm (3C), 100.5 ppm (9D), 29.3 ppm (4″E-N-CH3).

Dye 3 (Figure S7): ^1^H NMR (600 MHz, Methanol-d4) δ 7.81 ppm (d, J = 16.0 Hz, H-α), 7.79 ppm (d, J = 7.5 Hz, H-2′, H-6′), 7.51 ppm (d, J = 8.9 Hz, H-2″, H-6″), 7.51 ppm (m, H-4′), 7.47 ppm (d, J = 7.5 Hz, H-3′, H-5′), 7.41 ppm (d, J = 8.5 Hz, H-2‴, H-6‴), 7.39 ppm (s, H-6), 7.38 ppm (m, H-4‴), 7.26 ppm (d, J = 8.5 Hz, H-3‴, H-5‴), 7.23 ppm (s, H-8), 7.19 ppm (s, H-9), 7.11 ppm (s, H-3), 6.57 ppm (d, J = 16.0 Hz, H-β), 6.39 ppm (d, J = 8.0 Hz, H-3″, H-5″), 2.80 ppm (s, H-N-CH_3_). ^13^C NMR: δ 167.9 ppm (10D), 164.3 ppm (1-COO), 164.3 ppm (2C), 156.8 ppm (8aA), 150.0 ppm (7A), 148.6 ppm (C-H-α), 147.7 ppm (4″E), 133.0 ppm (4′B), 131.2 ppm (4‴F), 130.4 ppm (1″E), 130.8 ppm (2″, 6″), 129.2 ppm (1‴E), 128.6 ppm (3′, 5′B), 128.5 ppm (2‴, 6‴F), 126.8 ppm (2′, 6′B),126.7 ppm (1′B), 115.2 ppm (3‴, 5‴F), 112.6 ppm (3″, 5″E), 109.9 ppm (C-H-β), 108.7 ppm (4C), 106.4 ppm (4aA), 106.7 ppm (6A), 106.2 ppm (8A), 101.9 ppm (3C), 101.4 ppm (9D), 38.9 ppm (4″E-N-CH_3_).

The full assignment of NMR signals for dye 4 was more difficult due to the presence of a halogen in the structure. A low relaxation time led to an overlap of peaks and, consequently, a non-determination of the multiplicity. In this case, the analysis of two-dimensional techniques (COSY, HSQC, HMBC) was crucial for the assigned signals. Two different solvents were tested for this ester (deuterated methanol and dimethyl sulfoxide-d6), but the results were similar (Figure S7). ^1^H NMR (600 MHz, DMSO-d6) δ 7.96 ppm (H-α), 7.94 ppm (H-2′, H-6′), 7.65 ppm (H-2″, H-6″), 7.61 ppm (H-4′), 7.56 ppm (H-3′, H-5′), 7.54 ppm (H-2‴, H-6‴), 7.53 ppm (H-6), 7.49 ppm (H-4‴), 7.40 ppm (H-3‴, H-5‴), 7.38 ppm (H-8), 7.35 ppm (H-9), 7.27 ppm (H-3), 6.72 ppm (H-β), 6.54 ppm (H-3″, H-5″), 2.84 ppm (s, H-N-CH3). ^13^C NMR: δ 169.1 ppm (10D), 164.6 ppm (1-COO), 164.6 ppm (2C), 156.5 ppm (8aA), 155.6 ppm (1″E), 152.1 ppm (7A), 147.2 ppm (C-H-α), 134.0 ppm (4′B), 133.3 ppm (1‴E), 132.4 ppm (3‴, 5‴F), 131.6 ppm (2″, 6″), 131.2 ppm (2‴, 6‴F), 130.3 ppm (3″, 5″E),130.0 ppm (3′, 5′B), 127.6 ppm (1′B), 127.5 ppm (2′, 6′B), 125.5 ppm (4‴F), 117.5 ppm (C-H-β), 114.8 pm (4″E), 110.5 ppm (4C, 4aA), 107.6 ppm (6A, 8A), 103.4 ppm (3C), 102.4 ppm (9D), 40.3 ppm (4″E-N-CH_3_).

### 2.3. UV-Visible Spectroscopy 

**Figure 2:**
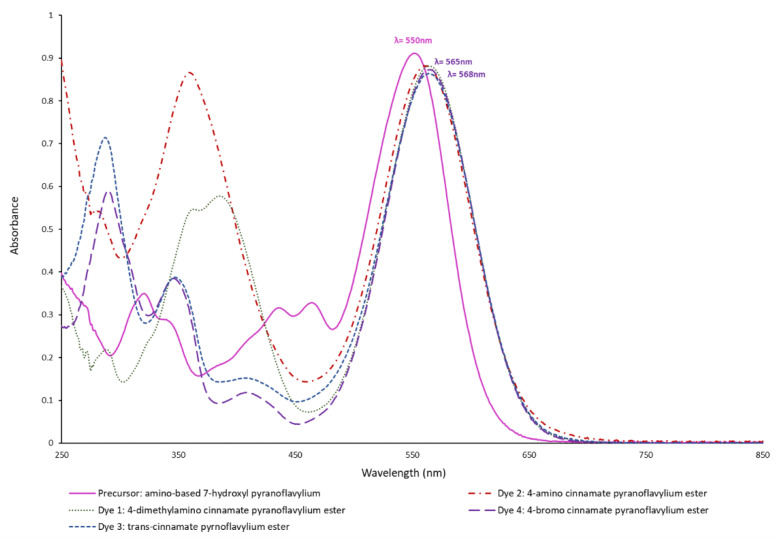
UV-Visible spectrum and maximum absorption wavelength for *p*-dimethylamino-phenyl-7-hydroxyl-pyranoflavylium (550 nm) and cinnamate pyranoflavylium esters (565 and 568 nm) recorded from the spectrophotometer in methanol.

UV-Visible spectra of these multi-chromophores showed that the inclusion of the cinnamic acid derivative in the pyranoflavylium structure produced a bathochromic shift, exhibiting a maximum absorption wavelength at 565 nm for the functionalization with 4-amino cinnamic acid and 568 nm for the functionalization with the other cinnamic acid derivatives, when compared to its pyranoflavylium precursor (550 nm) (Figure 2). As expected, the results did not show significant differences regardless of the substitution in the para position at the ring F of cinnamic acid, because the acceptor part and the cinnamic acid part are joined by the oxygen, a sp^3^ hybridized atom, making the whole molecule not π-conjugated. The main effect comes from removing the proton from the phenolic OH moiety. A redshift in the wavelength of maximum absorption was also reported in the anthocyanin’s esterification products prepared using enzymes [31,32].

The molar absorption coefficients (ε) of all compounds were also determined in four different solvents (methanol, ethanol, DMSO, and 20% (*v*/*v*) ethanol/water). It is well known that the nature of the solvent affects the solvatochromic characteristics of compounds (such as the wavelength of maximum absorption and the form and intensity of the absorption band) due to the intermolecular interactions with a solute. The values obtained for each compound are presented in Table 2, ranging from 6000 to 40,000 M^−1^·cm^−1^. The results showed that the remotion of the phenolic OH moiety affects the molar absorption coefficients. Regarding the functional group present in C-4‴ of ring F (N(CH_3_)_2_, NH_2_, H or Br), the ε values were higher in the presence of more electron donating groups—N(CH_3_)_2_ and NH_2_ (dye 1 and 2, respectively)—and decreased in the presence of a weak electron withdrawing group such as the halogen (Bromide—dye 4). Dyes 1 and 2 showed an important increase in the ε values when compared with the precursor, with particular emphasis on 20% (*v*/*v*) of ethanol/water. In contrast, dyes 3 and 4 showed a decrease in the ε values in all solvents, indicating that the presence of substituted amine groups has a positive impact on the molar absorption coefficients. When compared to the other solvents tested, all dyes displayed the lowest molar absorption coefficient values in 20% (*v*/*v*) ethanol/water, presumably indicating the presence of aggregation or self-association phenomena. In methanol, ethanol, and DMSO, the new dyes exhibit a high colorant capacity.

### 2.4. Fluorescence Quantum Yields

The determination of the fluorescence quantum yields was performed in polar aprotic (DMSO) and protic solvents (ethanol and methanol), and also in a binary mixture (aqueous solution 20% (*v*/*v*) ethanol/water). The values are present in Table 3 and demonstrated that the fluorescence quantum yields of the 7-hydroxyl-pyranoflavylium compound are not significantly affected by the functionalization of the compound. Low quantum yields were obtained for the pyranoflavylium precursor (<2%), and after functionalization, the values remained at ~1%. 

As complementary information, the fluorescence spectra in polar aprotic (DMSO) and protic solvents (ethanol and methanol), and also in a binary mixture (aqueous solution 20%(*v*/*v*) ethanol/water), were also investigated (Figure S8). Solutions were normalized to an absorbance of 0.1 to the maximum absorption wavelength of each compound (in the fluorescence cuvette) for all pigments. with excitation and emission slits set at 10 nm due to lower fluorescence quantum yields. The 7-hydroxy-pyranoflavylium precursor showed higher fluorescence intensities when compared with the pyranoflavylium-cinnamate esters, regardless of the solvent, which is in agreement with the literature and with the decrease in the fluorescence quantum yields (Table 3). The presence of a meta-directing group (-COOR), resulting from the esterification reaction, can decrease or even annul the fluorescence [33]. Regarding the solvent effect, the results showed that all the pigments had higher fluorescence in DMSO, with an emission maximum of around 663 nm for the 7-hydroxy-pyranoflavylium precursor and 658 nm for the new esters. The most likely explanation for the higher fluorescence intensities could be the high polarization character of DMSO (ε, dielectric constant of 46.7), which stabilizes the charge or dipole of a molecule [34]. In contrast, in 20% (*v*/*v*) ethanol/water, as expected, pigments showed a very weak fluorescence intensity, probably due to the aggregation or self-association phenomena that can occur in this mixture, as these compounds have a more hydrophobic character.

### 2.5. Acid-Base Reactions in Aqueous Solutions at Different pH Values

**Figure 3:**
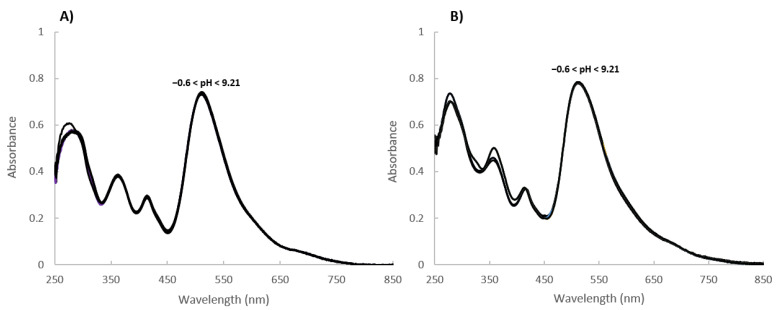
UV−Visible spectra of (**A**) trans-cinnamate pyranoflavylium ester (dye 3) and (**B**) 4-bromo cinnamate pyranoflavylium ester (dye 4) in aqueous solution at different pHs.

The acid-base reactions and respective equilibrium forms of the different cinnamate pyranoflavylium esters were evaluated in an aqueous solution (20% ethanol in water) at different pH values (−0.6, −0.3, 0, 1, 1.5, 2.19, 3.09, 5.12, 7.15, and 9.21) by UV-Visible spectroscopy. As expected, based on the structure of the pigment, the UV-Visible spectrum showed important modifications at very acidic pH values, resulting from the protonation of the tertiary amine group present in ring E. In synthetic flavylium/pyranoflavylium compounds bearing amino substituents, it has been reported that the amino group can be protonated at very low pH values in the range of 0 < pH < −0.8 [35]. Contrarily to anthocyanins and other flavylium dyes, pyranoanthocyanins are protected from the nucleophilic attack by water and, because of this, do not consequently undergo hydration reactions. Also, the pigments are not converted into hemiketal form to give *cis*- or *trans*-chalcone by tautomerization [10,12]. Furthermore, the absence of hydroxyl groups in the cinnamate ester structure prevents the occurrence of a deprotonation reaction on the flavylium core to yield the respective quinoidal form. As presented in Figure 3, the *trans* and 4-bromo cinnamate pyranoflavylium esters (dyes 3 and 4, respectively) present overlapped spectra at different pH values, with a maximum absorption wavelength of 512 nm. Even in very acidic conditions (proton concentrations of 4 M and 2 M), the modification in the spectrum was not observed, which indicates that the protonation of the amino group in ring E of pyranoflavylium core requires extremely acidic conditions (HCl concentrations of 5 M and/or 6 M), as reported in the literature [36]. By this, for these two new esters, regardless of the pH value, the flavylium cation (AH^+^) is the only specie present.

**Figure 4:**
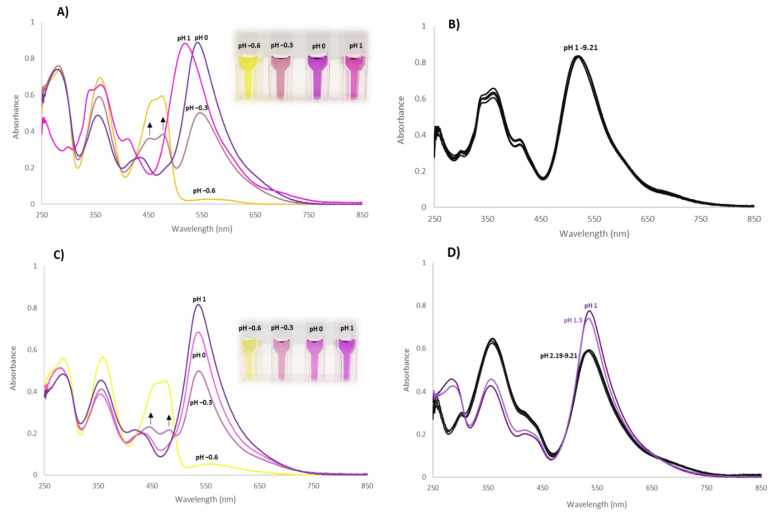
UV-Visible spectra of 4-dimethylamino cinnamate pyranoflavylium ester in aqueous solution at (**A**) pH −0.6, −0.3, 0, and 1, and (**B**) pH range of 1–9.21, and 4-amino cinnamate pyranoflavylium ester in aqueous solutions at (**C**) pH −0.6, −0.3, 0, and 1 and (**D**) pH range of 1–9.21.

Regarding 4-dimethylamino and 4-amino cinnamate pyranoflavylium esters (dyes 1 and 2, respectively) in the same conditions, the UV-Visible spectrum showed some modifications due to the occurrence of the protonation of amino groups. As shown in Figure 4A, in the case of dye 1, the pigment showed a maximum absorbance wavelength at 523 nm at pH 1 and, at pH 0, a bathochromic shift of 26 nm is observed, which can correspond to the protonation of the secondary amine in ring E, resulting in a dicationic species (AH^+^-NHR_2_^+^) (Figure 4C). Until pH −0.6, the results showed a decrease in the absorbance intensity for the dicationic species and the appearance of two shoulders at 456 and 479 nm as a result of a possible protonation of the secondary amine present in ring F (tricationic specie, AH^+^-NHR_2_^+^-NHR_2_^+^). For higher pH values, between 1 and 9.21 (Figure 4B), the pigment showed an overlapping UV-Vis spectrum, which means that no proton transfer occurs, and a maximum absorbance wavelength at 523 nm is observed. 

As to the UV-Visible spectrum of dye 2, the results were slightly different when compared with dye 1. In this case, at pH 1, the cationic specie (AH^+^) presents a maximum absorbance wavelength at 538 nm, and for pH 0, the intensity of this specie decreases, which can indicate the protonation of the secondary amine in group E (dicationic specie, AH^+^-NHR_2_^+^). In more acidic conditions, the second protonation is visible at pH −0.3 with the appearance of two shoulders at 447 and 476 nm, and until pH −0.6, the cation flavylium specie ends up disappearing as a result of the maximum increase in the tricationic specie (AH^+^-NHR_2_^+^-NHR_2_^+^), resulting from the complete protonation of the amine group present in ring F. This hypsochromic shift is the typical behavior described in the literature for these types of pigments in acidic conditions [10,36,37], conferring different colors to the pigment in aqueous solutions, resulting in the proton transfer reactions. For a pH range between 2.19 and 9.21 (Figure 4D), the pigment showed an overlapped UV-Visible spectrum with two maximum absorption wavelengths at 538 and 363 nm. 

The protonation of amine groups in dyes 1 and 2 occurs at higher pH values when compared with dyes 3 and 4, and for pH 1–9, the cinnamate pyranoflavylium esters do not undergo proton transfer processes, maintaining the same species in solution. This work reports, for the first time, the synthesis of pyranoflavylium dyes with extended chromatic stability through functionalization of the hydroxyl group with different cinnamic acid derivatives using EDC coupling chemistry. 

### 2.6. Dyes Stability over Time at Different pH Values

**Figure 5:**
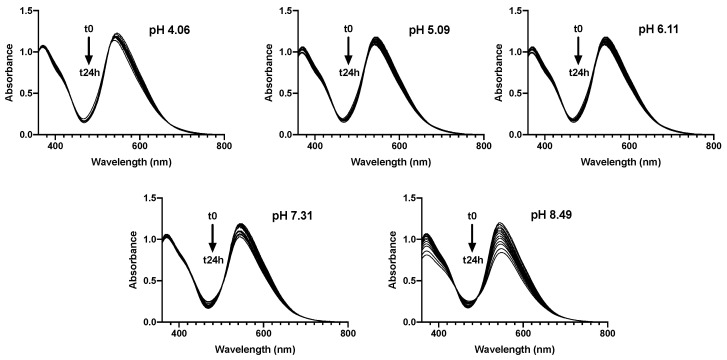
UV-Visible spectra of dye 1 (4-dimethylamino pyranoflavylium cinnamate ester) in aqueous solution with 50% (*v*/*v*) ethanol/water at different pH values from 4 to 8 over time. The spectra were recorded for 24 h at different pH values.

Considering that ester functionality is naturally labile toward hydrolysis, particularly at high and low pH ranges, the synthesized dyes’ stability over time was investigated in aqueous media at different pH values (pH 4 to 8). These preliminary studies were only performed for dye 1 (4-dimethylamino cinnamates ester) at different pH values by UV-Visible spectroscopy. The study was performed in a pH range between 4 and 8, due to the potential applications of these dyes, using 50% (*v*/*v*) of ethanol/water in the final solution. Given that these pigments are mostly hydrophobic, when lower concentrations of ethanol (for example, 20% *v*/*v*) were used, dye aggregation and precipitation after 1 h at room temperature were observed. In 50% (*v*/*v*) ethanol/water, for pH 4.06, 5.09, and 6.11, it can be observed that the dye is stable during the 24 h analyzed (Figure 5), only undergoing a minor decrease in the intensity at the maximum absorption wavelength, which may be due to the dye’s aggregation. The stability of the dye at pH 4–6 during 24 h is also corroborated with the HPLC chromatograms—Figure S9, which shows that only the dye is present and no peak corresponding to the 7-hydroxy-pyranoflavylium precursor is observed. Regarding pH 7.31 and 8.49, this decrease was more pronounced over time. At pH 8.49, in addition to the important decrease in the intensity at the maximum absorption wavelength, the spectrum showed a hypsochromic shift in the maximum absorption wavelength. As expected for higher pH values, this is the result of the hydrolysis of the ester bond. In the HPLC chromatogram, it is evident that the appearance of a new peak corresponds to the 7-hydroxyl-pyranoflavylium precursor at a retention time of 16 min. This hydrolysis was more evident at pH 8.49 (~52%) when compared to pH 7.31 (~31%). 

## 3. Materials and Methods

### 3.1. General Information

*Trans*-cinnamic acid (≥98.0%) and 4-bromo cinnamic acid were purchased from Acros Organics. 4-amino cinnamic acid and 4-dimethylamino cinnamic acid were purchased from Sigma Aldrich. 1-(3-dimethylaminopropyl)-3-ethylcarbodiimide hydrochloride (EDC, ≥98%, Germany) was obtained from Alfa Aesar. 4-(Dimethylamino) pyridine ≥99% was purchased from Sigma-Aldrich. The solvents ethanol (99.5%) and dimethylformamide were purchased from Aga and VWR, respectively. The 7-hydroxy-10-(4″-dimethylamino-cinnamyl)-pyranoflavylium pigment was synthesized according to the procedure described by Chassaing et al.: through the condensation of a 4-methylflavylium salt with 4-dimethylaminobenzaldehyde [14]. 

### 3.2. General Synthesis of Pyranoflavylium-Cinnamate Esters

The esterification reaction of 7-hydroxyl-4″-(dimethylamino)-cinnamyl-10-pyranoflavylium with different cinnamic acids was optimized using different experimental conditions (Table 1 and Table S1). In general, and as an example for one of the cinnamic acids used, in the best reaction conditions, 4-dimethylamino cinnamic acid (8.36 mg, 4.36 × 10^−5^ mol) was dissolved in the organic solvent (dimethylformamide, DMF, 1 mL) and, then, the coupling agent, 1-(3-dimethylaminopropyl)-3-ethylcarbodiimide hydrochloride (33.98 mg, 2.18 × 10^−4^ mol, EDC), was added to the solution. After complete dissolution, the 7-hydroxy-pyranoflavylium pigment (1.67 mg, 4.37 × 10^−6^ mol) was added to the activated cinnamic acid, and the mixture was left to react at room temperature in the dark under stirring for 1 h. The formation of the pyranoflavylium cinnamate ester was monitored by HPLC-DAD analysis. After reaching the maximum formation of the ester, the reaction was stopped by the addition of water and, then, the mixture was purified using a Büchner funnel system with a porous plaque (porosity 3) under vacuum using RP-18 (40–63 μm, Merck, Germany) gel as stationary phase. The esterified pyranoflavylium compound was recovered with 80% (*v*/*v*) methanol. The novel functionalized pigments were freeze-dried and stored at −20 °C until further analysis.

### 3.3. HPLC/LC-MS Analysis 

The esterification reaction was monitored by HPLC-DAD (Merck) in a reverse-phase C18 column (Agilent) with 250 × 4.6 mm i.d., 2.7 μm at 25 °C. The eluents used were (A) 1% (*v*/*v*) formic acid in water and (B) 1% (*v*/*v*) formic acid in acetonitrile, and the elution gradient was performed from 50 to 100% B during 51 min at a flow rate of 0.4 mL/min. After 51 min, the column was washed with 100% B for 15 min and, then, it was stabilized with the initial conditions for more 10 min. 

Mass spectrometry (MS) analysis of the pyranoflavylium-cinnamic derivative esters was performed using a Finnigan Surveyor series liquid chromatograph equipped with a reversed-phase C18 column (Agilent) with 250 × 4.6 mm i.d., 2.7 μm thermostatted at 25 °C). The mass detection was carried out in the positive ion mode in a Finnigan LCQ DECA XP MAX (Finnigan Corp., San José, CA, USA) mass detector with an API (Atmospheric Pressure Ionization) source of ionization and an ESI (ElectroSpray Ionization) interface. The solvents and HPLC gradient were used as the same reported above for the HPLC analysis. Spectra were recorded in the positive ion mode between *m/z* 300 and 1500.

### 3.4. NMR Spectroscopy 

The full structural characterization of ester products was performed on a Bruker-Avance III HD 600 MHz 14.1 Tesla spectrometer using 1D (^1^H) and 2D (COSY, gHSQC and gHMBC) NMR experiments in deuterated methanol (CD_3_OD) (esters from trans-, 4-amino and 4-dimethylamino cinnamic acids) and deuterated dimethyl sulfoxide (DMSO-d6) for the ester obtained from 4-bromo cinnamic acid. ^1^H chemical shifts were assigned using a 2D NMR (COSY) experiment, while the ^13^C resonances were assigned using 2D NMR techniques (gHMBC and gHSQC).

### 3.5. Determination of Molar Absorption Coefficients (ε)

The molar absorption coefficient (ε) of the new pigments, and also the 7-hydroxy- pyranoflavylium precursor, were determined in four solvents, namely methanol, ethanol, dimethyl sulfoxide (DMSO), and an aqueous solution of 20% (*v*/*v*) ethanol/water. In each solvent, the respective pigment was prepared at different concentrations and, then, the absorbance at the maximum absorption wavelength (determined using the most concentrated solution of each dye) was obtained for each solution. The respective absorbances were represented as a function of dye concentration, and the ε value was determined from the slope value of the straight equation because it used a 1 cm optical path quartz cell. 

### 3.6. Fluorescence Quantum Yields

Based on the absorbance maxima of the different compounds, the determination of the quantum yield (QY) of the new synthesized esters, and the 7-hydroxy-pyranoflavylium precursor, was performed using a calibrated Quantaurus-QY stand-alone integrating setup C11347-11 (Hamamatsu Photonics, Hamamatsu, Japan) equipped with an integrating sphere, a monochromator, a spectrograph, a 150 W xenon light source, and a silicon charge-coupled device. Each compound was dissolved in four different solvents (ethanol, methanol, DMSO, and 20% (*v*/*v*) ethanol/water) and diluted to ensure an absorbance of 0.1 from the wavelength of forward excitation. Three independent experiments were performed for each component.

### 3.7. Titration of the Cinnamate Ester Pigments by UV-Visible Spectroscopy

Stock solutions of the different pyranoflavylium cinnamate esters (1.25 × 10^−4^ M) were prepared in ethanol to obtain a concentration of 20% (*v*/*v*) ethanol/water in the final solution. Within a 10 mm × 10 mm quartz cell, 500 µL of universal buffer at the correspondent pH (2, 3, 5, 7, and 9), HCl solution at 4 M, 2 M, 1 M, or 0.1 M, 700 µL of water, and 300 µL of pigment sock solution (final concentration of 2.5 × 10^−5^ M) were added. For each pigment, the first spectrum (250–800 nm) was recorded immediately after the addition of the pigment stock solution and shaking. pH values of the final solutions (pH of 2.19, 3.09, 5.12, 7.15, and 9.21) were checked in a WTW (pH 320 or 508) (Weilheim, Germany) with a CRISON 5209 combined glass electrode of 3 mm diameter (Barcelona, Spain), previously calibrated with buffer solutions (pH 4, 7 and 10). The stability of the ester bond towards hydrolysis was also studied by UV-Visible spectroscopy at different pH values, ranging from 4 to 8 over time in 50% (*v*/*v*) ethanol/water.

## 4. Conclusions

This work reports a fast, environmentally friendly, and effective alternative for the functionalizations of flavylium/pyranoflavylium dyes via coupling chemistry with EDC. The esterification of several cinnamic acids with a 7-hydroxyl pyranoflavylium has been described, and it was carried out in a reactional step with excellent reaction yields (60–99%). The results showed a bathochromic shift in the maximum absorption wavelength of the cinnamate pyranoflavylium esters and that the presence of more powerful electron-donating groups (NH_2_ and N(CH_3_)_2_) yielded a higher molar absorption coefficient when compared with dyes presenting electron-withdrawing groups (Br) in the para position of the aromatic ring in cinnamic structure. The UV-Visible spectra at different pH values confirmed no proton transfer reactions were involved after functionalization of the hydroxyl group at the C-7 of ring A in pyranoflavylium structure, and contrarily to the precursor, for a pH range between 2 and 9, only one species is present in solution. To conclude, it should be highlighted that this work demonstrates that the functionalization of anthocyanin derivatives prevents the occurrence of proton transfer reactions and enhances the color stability of dyes at a wide pH range, especially from pH 4 to 6. Additional studies regarding their biological properties, namely antioxidant, antimicrobial, and the ability to inhibit some skin-related enzymes, will be performed for putative incorporation of these dyes into lipophilic matrices for therapeutic and cosmetic purposes.

## Figures and Tables

**Table 1:** Optimization of the reaction conditions for *p*-dimethylamino-phenyl-7-hydroxyl pyranoflavylium functionalization with 4-dimethylamino cinnamic acid.

Entry	DMAC: EDC	DMAC: 7-Hydroxy-pyranoflavylium	EDC: DMAP	Solvent	Temperature	Reaction Time	Reaction Yield
1	-	(3:1)	-	DMF	r.t.	2 h	No product
2	(1:2)	(5:1)	-	Ethanol	r.t	*(a)* 1 h	No product
						*(b)* 4 days	No product
3	(1:2)	(5:1)	-	Ethanol	Reflux	16 h	No product
4	(1:2)	(5:1)	-	DMF	r.t.	*(a)* 1 h	24%
						*(b)* 3 h	41%
						*(c)* 7 h	85%
						*(d)* 24 h	87%
5	(1:10)	(10:1)	-	DMF	r.t.	*(a)* 1 h	85%
						*(b)* 3 h	94%
6	(1:5)	(10:1)	-	DMF	r.t.	1 h	99%
7	(1:3)	(10:1)	-	DMF	r.t.	*(a)* 1 h	55%
						*b)* 5 h	99%
8	(1:2)	(10:1)	-	DMF	r.t.	*(a)* 1 h	5%
						*(b)* 3 h	28%
						*(c)* 7 h	37%
						*(d)* 24 h	42%
9	(1:1)	(10:1)	-	DMF	r.t.	*(a)* 1 h	8%
						*(b)* 3 h	16%
						*(c)* 7 h	25%
						*(d)* 24 h	24%
10	(1:2)	(10:1)	(1:1)	DMF	r.t	*(a)* 1 h	49%
						*(b)* 6 h	19%
11	(1:5)	(5:1)	-	DMF	r.t.	*(a)* 1 h	44%
						*(b)* 5 h	72%
						*(c)* 20 h	99%

DMF—dimethylformamide, r.t. room temperature.

**Table 2:** Molar absorption coefficients (ε) of all new esters in four different solvents (methanol, ethanol, DMSO, and 20% (*v*/*v*) ethanol/water). Counter-ion: chloride.

Dye	ε (M^−1^·cm^−1^)			
	Methanol	Ethanol	DMSO	20% (*v*/*v*) Ethanol/Water
7-hydroxyl pyranoflavylium	3.5 × 10^4^ (551 nm)	3.6 × 10^4^ (554 nm)	3.2 × 10^4^ (560 nm)	1.3 × 10^4^ (546 nm)
Dye 1	3.9 × 10^4^ (563 nm)	3.7 × 10^4^ (568 nm)	3.4 × 10^4^ (576 nm)	2.4 × 10^4^ (525 nm)
Dye 2	3.5 × 10^4^ (566 nm)	3.7 × 10^4^ (565 nm)	3.0 × 10^4^ (567 nm)	1.7 × 10^4^ (537 nm)
Dye 3	2.5 × 10^4^ (565 nm)	2.6 × 10^4^ (568 nm)	9.0 × 10^3^ (573 nm)	6.0 × 10^3^ (528 nm)
Dye 4	2.4 × 10^4^ (566 nm)	2.6 × 10^4^ (570 nm)	2.8 × 10^4^ (576 nm)	9.2 × 10^3^ (506 nm)

**Table 3:** Fluorescence quantum yields for the different compounds determined by the absolute method using an integrating sphere.

Compound	Fluorescence Quantum Yields ± SD (%)			
	DMSO	Methanol	Ethanol	20%Ethanol/Water (*v*/*v*)
7-hydroxyl pyranoflavylium	(1.47 ± 0.21)	(1.50 ± 0.24)	(1.80 ± 0.08)	(0.87 ± 0.12)
Dye 1	(1.40 ± 0.14)	(1.20 ± 0.14)	(1.13 ± 0.12) ****	(1.00 ± 0.08)
Dye 2	(1.20 ± 0.22)	(0.97 ± 0.05) *	(0.77 ± 0.05) ****	(0.80 ± 0.08)
Dye 3	(1.03 ± 0.17)	(0.97 ± 0.12) *	(0.80 ± 0.08) ****	(0.47 ± 0.09) *
Dye 4	(0.90 ± 0.08)	(0.90 ± 0.08) *	(0.93 ± 0.05) ****	(0.70 ± 0.14)

* Asterisks were used to indicate significance values between 7-hydroxyl-pyranoflavylium precursor and the new dyes: * *p* < 0.05; **** *p* < 0.001.

## Data Availability

The data presented in this study are available on request from the corresponding author.

## Supplementary Materials

The following supporting information can be downloaded at: https://www.mdpi.com/article/10.3390/molecules27217351/s1. Table S1: Optimization of the reaction conditions for *p*-dimethylamino-phenyl-7-hydroxyl pyranoflavylium functionalization with trans-cinnamic acid, 4-bromo, and 4-dimethylamino cinnamic acid. Scheme S1: Nucleophilic addition of a primary amine present in dye 2 to the carbonyl group of the solvent (DMF), resulting in a side-product with [M+] *m/z* 582 and a maximum absorption wavelength of 570 nm. Figure S1: HPLC chromatograms obtained at a wavelength maximum of each dye. Dye 1 (A), dye 2 (B), dye 3 (C) and dye 4 (D). Figure S2: HRMS analysis performed in the positive-ion mode of the new ester compound formed during the esterification reaction of amino-based 7-hydroxyl-pyranoflavylium and 4-dimethyl amino cinnamic acid. Figure S3: HRMS analysis performed in the positive-ion mode of the new ester compound formed during the esterification reaction of amino-based 7-hydroxyl-pyranoflavylium and 4-amino cinnamic acid. Figure S4: HRMS analysis performed in the positive-ion mode of the new ester compound formed during the esterification reaction of amino-based 7-hydroxyl-pyranoflavylium and trans-cinnamic acid. Figure S5: HRMS analysis performed in the positive-ion mode of the new ester compound formed during the esterification reaction of amino-based 7-hydroxyl-pyranoflavylium and 4-bromo cinnamic acid. Figure S6: ^1^H-NMR spectrum in deuterated methanol of 4-dimethylamino cinnamate pyranoflavylium ester (Dye 1). Figure S7: ^1^H-NMR spectra in deuterated methanol of 4-amino cinnamate pyranoflavylium ester (Dye 2—blue), trans-cinnamate pyranoflavylium ester (dye 3—red), and 4-bromo cinnamate pyranoflavylium ester (Dye 4—green). Figure S8: Fluorescence spectra for the pigments in different solvents. Pink: 7-hydroxyl pyranoflavylium precursor. Purple: dye 1; green: dye 2; blue: dye 3; and black: dye 4. Figure S9: HPLC chromatograms at 566 nm for dye 1 (4-dimethylamino pyranoflavylium cinnamate ester) in aqueous solution with 50% (*v*/*v*) ethanol/water at different pH values after 27 h of incubation at room temperature.
