# Supplementary material for: Functionalization of 7-Hydroxy-pyranoflavylium: Synthesis of New Dyes with Extended Chromatic Stability
Source: Molecules. 2022 Oct 29;27(21):7351. doi: 10.3390/molecules27217351 (PMC9657386; doi:10.3390/molecules27217351)
Supplement: Supplementary file 1 [file molecules-27-07351-s001.zip › molecules-1963883-supplementary.pdf]

Supplementary material

Functionalization of 7-hydroxy-pyranoflavylium: synthesis of new dyes with extended chromatic stability.

Ana Rita Pereira, Victor de Freitas, Nuno Mateus*, and Joana Oliveira*

Laboratório Associado para a Química Verde – REQUIMTE, Departamento de Química e Bioquímica, Faculdade de Ciências, Universidade do Porto, Rua do Campo Alegre, 687, 4169-007 Porto, Portugal
* nbmateus@fc.up.pt (N.M.); * jsoliveira@fc.up.pt (J.O.).

Keywords: 7-hydroxy-pyranoflavylium; 1-(3-dimethylaminopropyl)-3-ethylcarbodiimide hydrochloride; ester bond; coupling chemistry; cinnamic acids

Table S1: Optimization of the reaction conditions for p-dimethylamino-phenyl-7-hydroxyl pyranoflavylium functionalization with trans-cinnamic acid, 4-bromo, and 4-dimethylamino cinnamic acid (DMF – dimethylformamide, r.t. room temperature).

Entry	COOH: EDC	COOH: OH	EDC: DMAP	Solvent	Temperature	Reaction Time	Reaction yield
**Functionalization with trans-cinnamic acid**							
1	(1:2)	(10:1)	-	DMF	r.t.	a) 1 h	45%
						b) 17 h	83%
2	(1:3)	(10:1)	-	DMF	r.t.	a) 1 h	28%
						b) 4 h	85%
						c) 7 h	65%
3	(1:3)	(5:1)	-	DMF	r.t.	a) 1 h	32%
						b) 7 h	44%
						c) 23 h	44%
						d) 48 h	43%
**Functionalization with 4-bromo cinnamic acid**							
4	(1:2)	(10:1)	-	DMF	r.t.	a) 1 h	46%
						b) 17 h	95%
5	(1:3)	(10:1)	-	DMF	r.t.	a) 1 h	59%
						b) 4 h	70%
						c) 7 h	93%
6	(1:2)	(5:1)	-	DMF	r.t.	a) 1 h	41%
						b) 4 h	57%
						c) 24 h	60%
**Functionalization with 4-amino cinnamic acid**							
7	(1:2)	(10:1)	-	DMF	r.t.	1 h	67%
8	(1:1)	(10:1)	-	DMF	r.t.	a) 1 h	42%
						b) 6 h	69%
9	(1:5)	(5:1)	-	DMF	r.t.	a) 1 h	3%
						b) 7 h	32%
						c) 24 h	67%

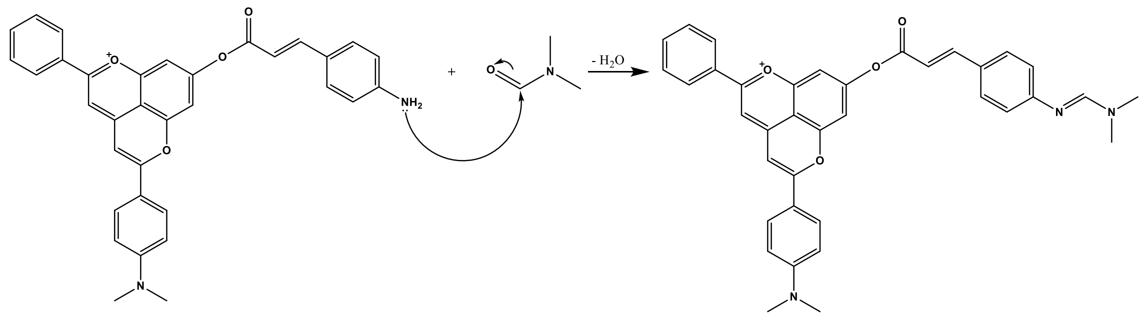

Scheme S1: Nucleophilic addition of a primary amine present in dye 2 to the carbonyl group of the solvent (DMF) resulting in a side-product with [M^+] *m*/*z* 582 and a maximum absorption wavelength of 570 nm.

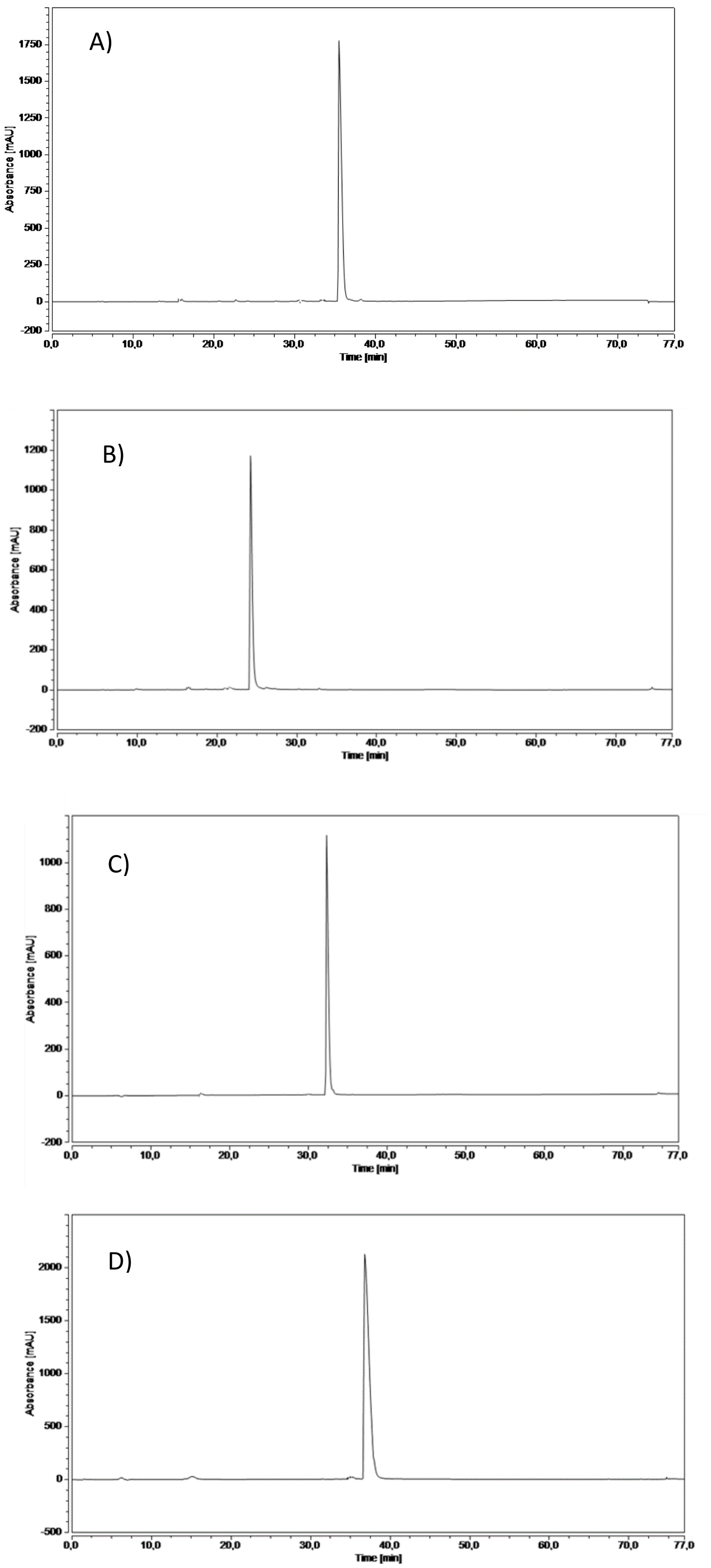

Figure S1 - HPLC chromatograms obtained at a wavelength maximum of each dye. Dye 1 (A), Dye 2 (B), dye 3 (C) and dye 4 (D).

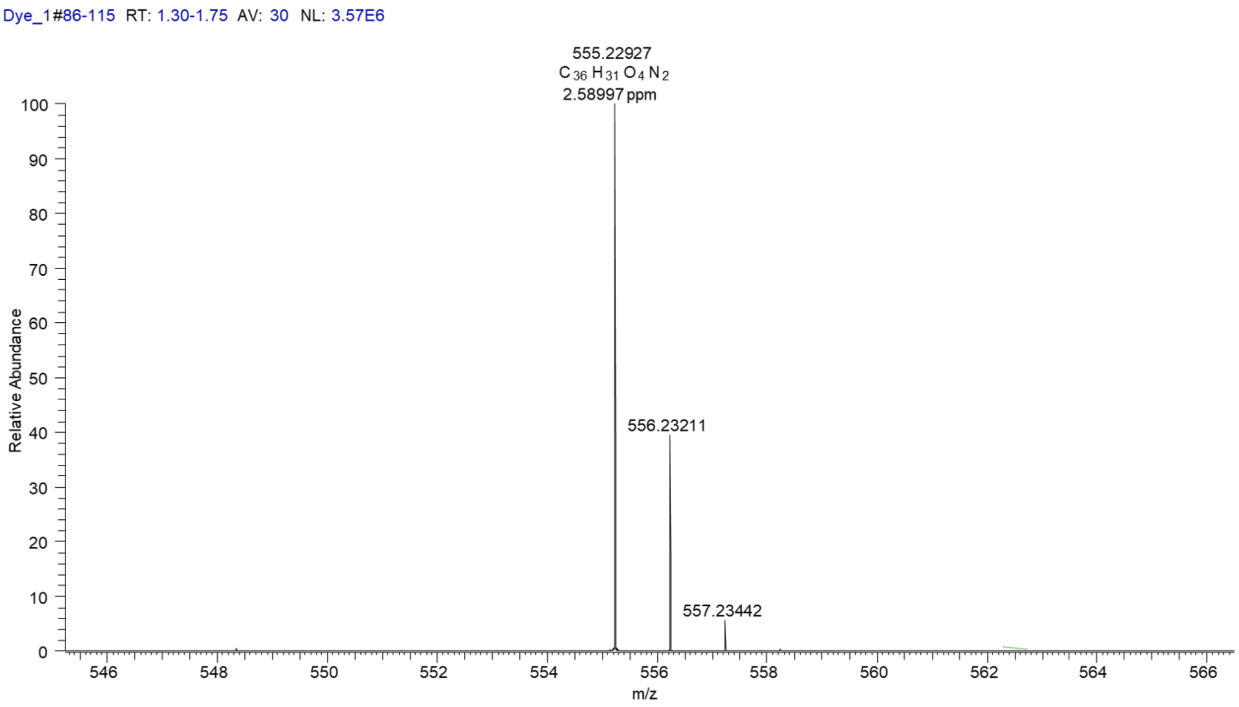

Figure S2: HRMS analysis performed in the positive-ion mode of the new ester compound formed during the esterification reaction of amino-based 7-hydroxyl-pyranoflavylium and 4-dimethyl amino cinnamic acid.

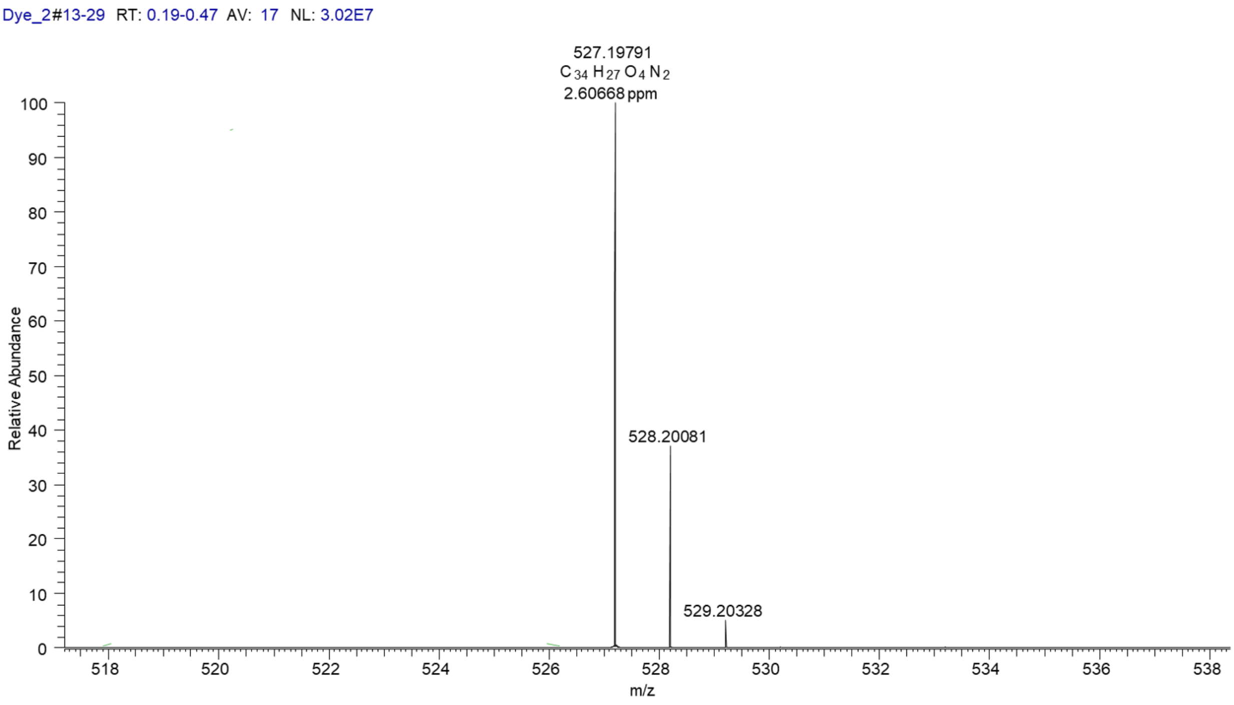

Figure S3: HRMS analysis performed in the positive-ion mode of the new ester compound formed during the esterification reaction of amino-based 7-hydroxyl-pyranoflavylium and 4-amino cinnamic acid.

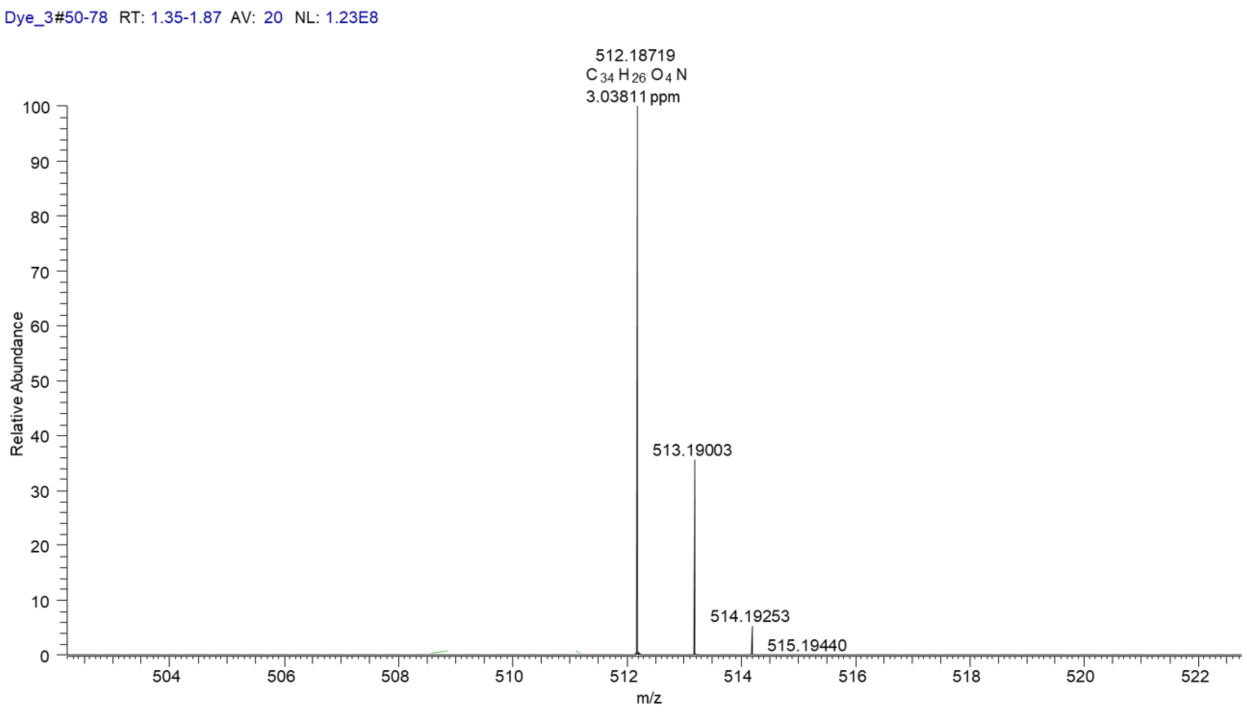

Figure S4: HRMS analysis performed in the positive-ion mode of the new ester compound formed during the esterification reaction of amino-based 7-hydroxyl-pyranoflavylium and trans-cinnamic acid.

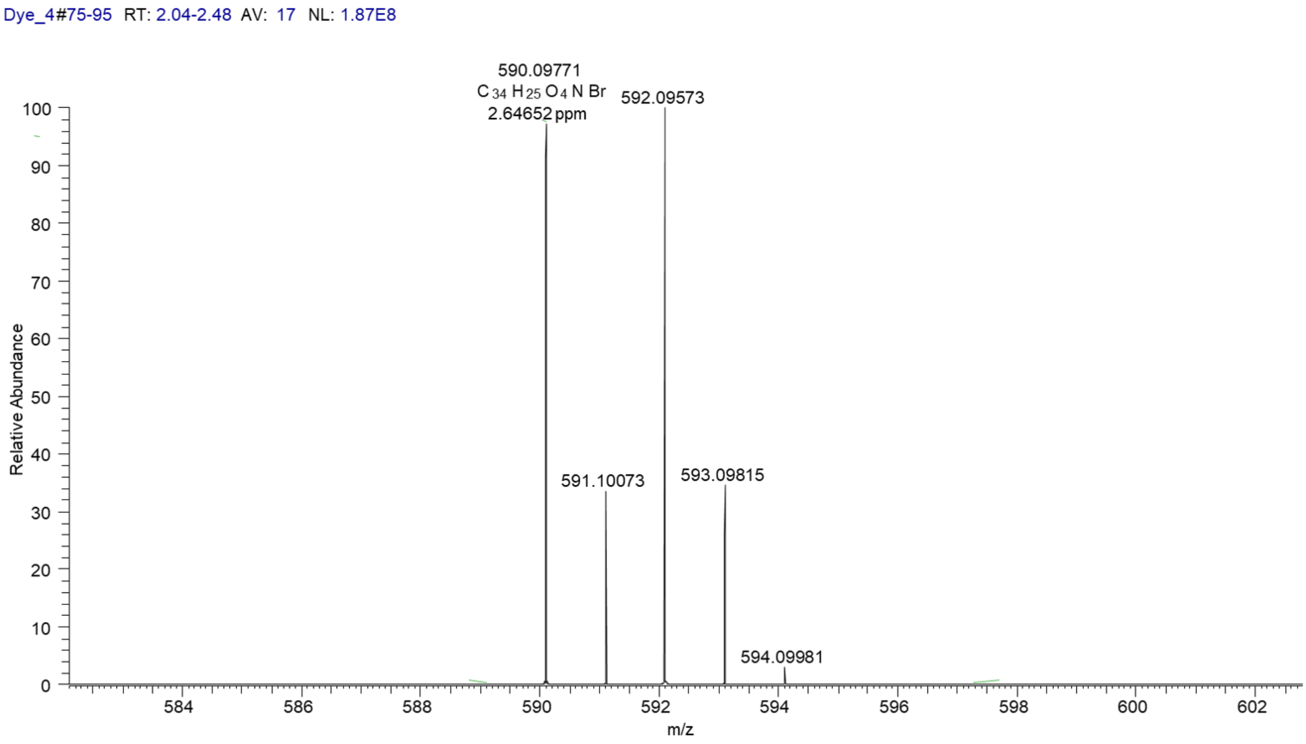

Figure S5: HRMS analysis performed in the positive-ion mode of the new ester compound formed during the esterification reaction of amino-based 7-hydroxyl-pyranoflavylium and 4-bromo cinnamic acid.

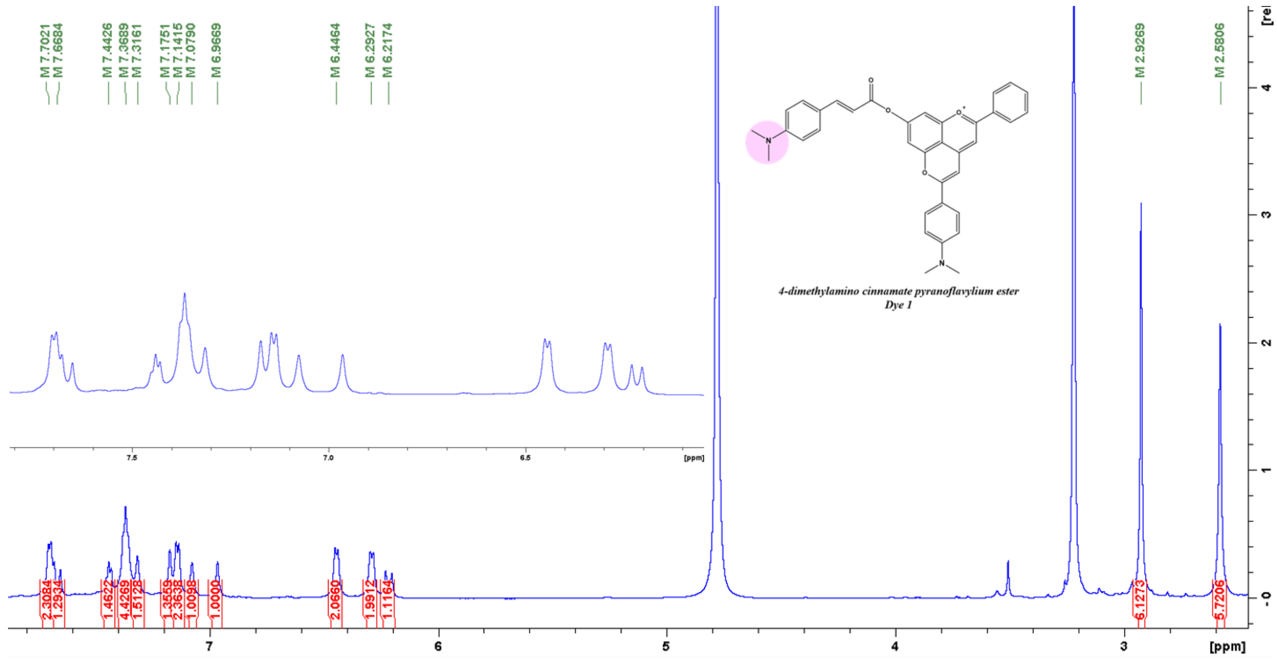

Figure S6 - ¹H-NMR spectrum in methanol deuterated of 4-dimethylamino cinnamate pyranoflavylium ester (Dye 1)

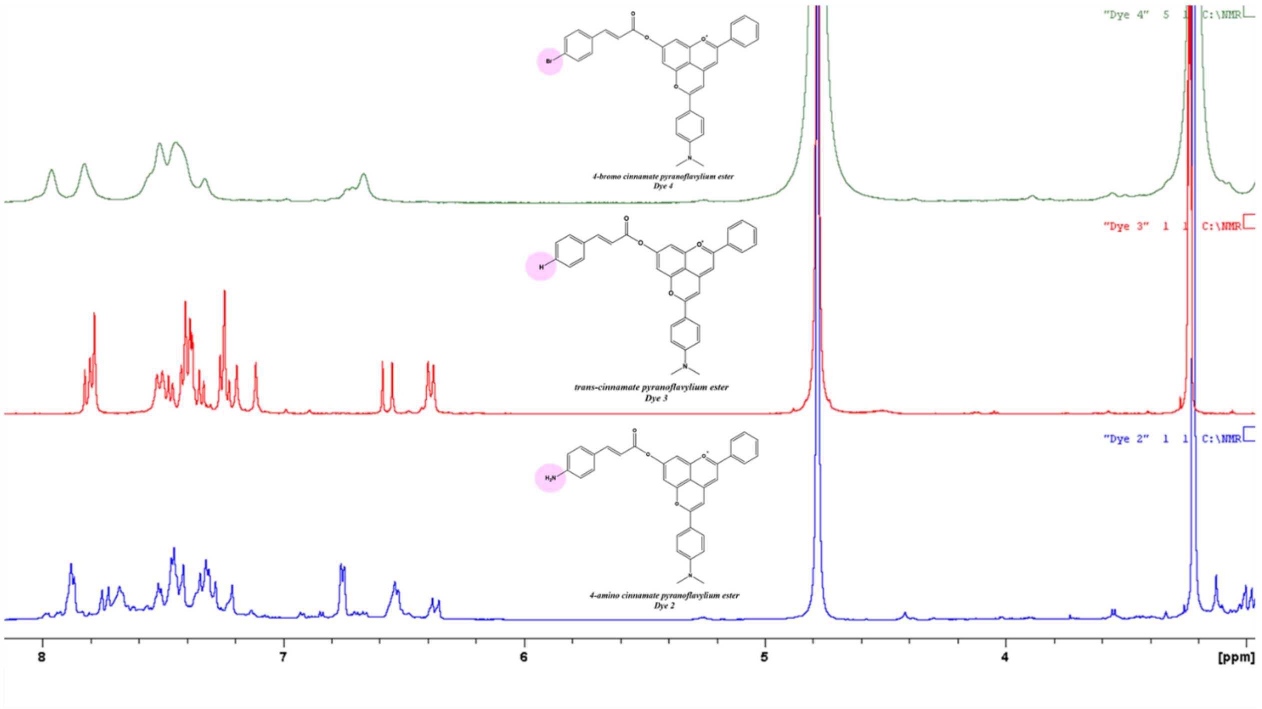

Figure S7 - ¹H NMR spectra in methanol deuterated of 4-amino cinnamate pyranoflavylium ester (Dye 2 - blue), *trans*-cinnamate pyranoflavylium ester (Dye 3 - red) and 4-bromo cinnamate pyranoflavylium ester (Dye 4 - green)

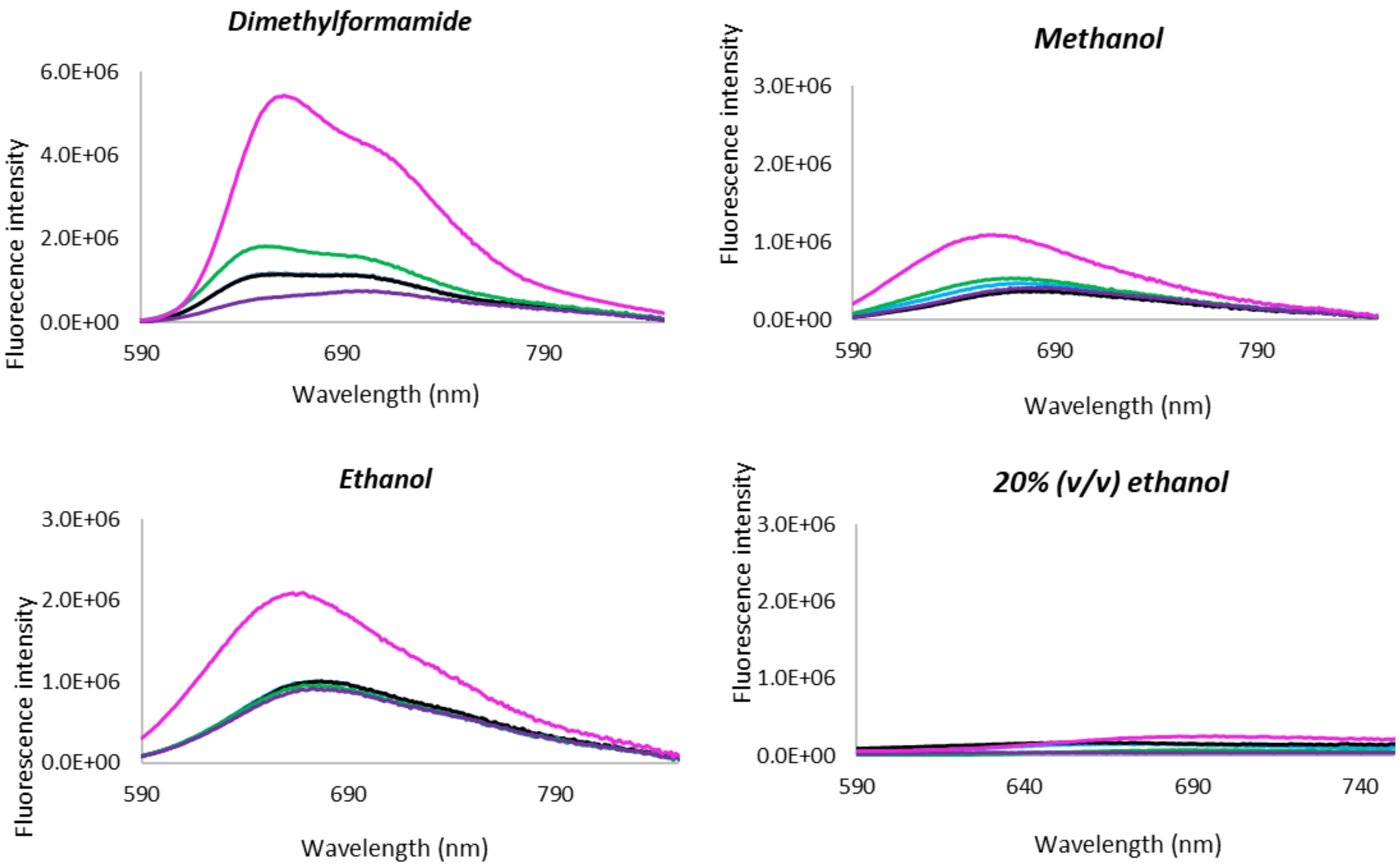

Figure S8 - Fluorescence spectra for the pigments in different solvents. Pink: 7-hydroxyl pyranoflavylium precursor. Purple: dye 1; Green: dye 2; Blue: dye 3 and Black: dye 4.

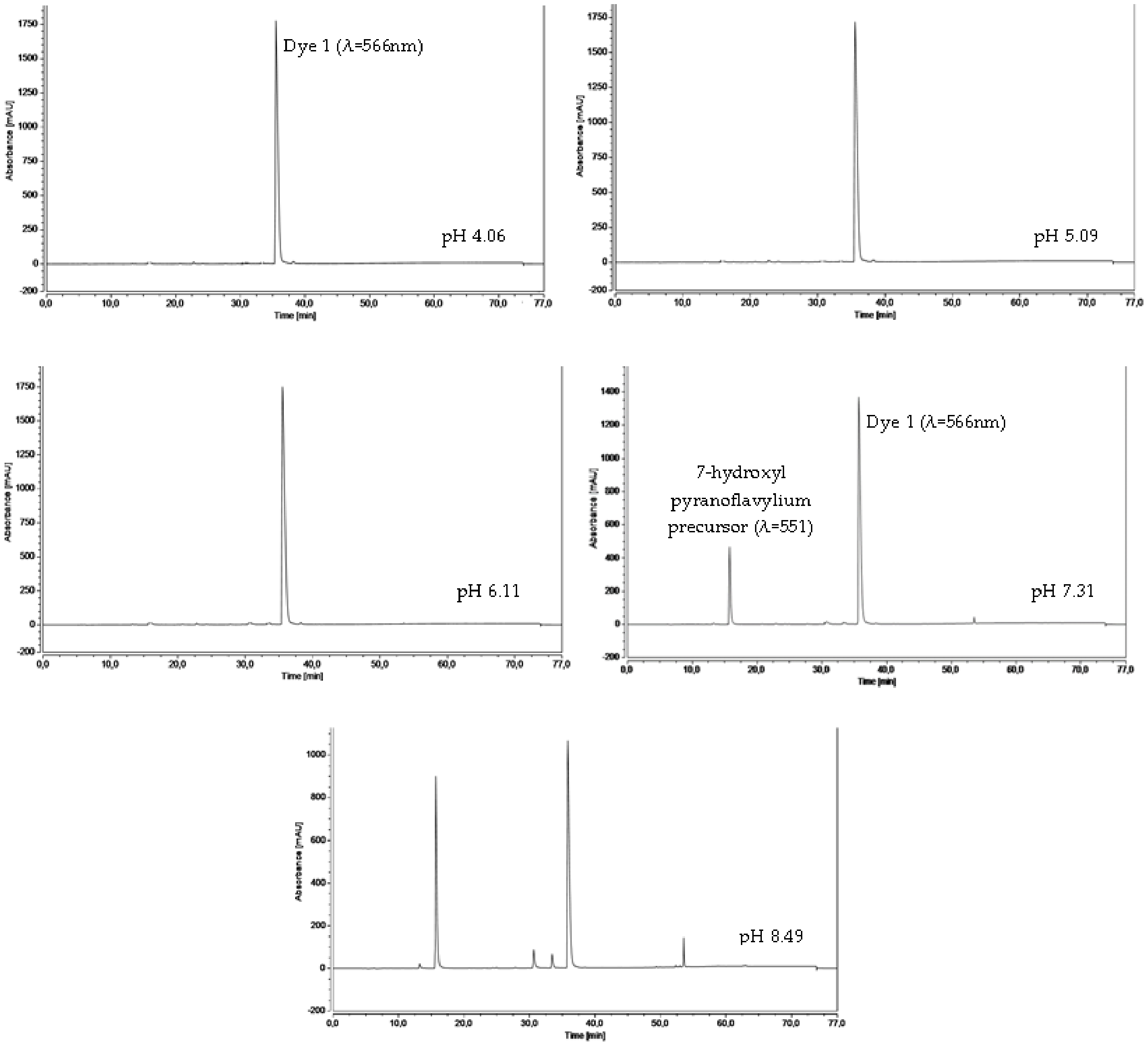

Figure S9 – HPLC chromatograms for dye 1 (4-dimethylamino pyranoflavylium cinnamate ester) in aqueous solution with 50% (v/v) ethanol/water at different pH values after 27 hours at room temperature.
